# Response to anti-tuberculosis treatment by people over age 60 in Kampala, Uganda

**DOI:** 10.1371/journal.pone.0208390

**Published:** 2018-12-19

**Authors:** Nicholas Sebuliba Kirirabwa, Derrick Kimuli, Seyoum DeJene, Carol Nanziri, Estella Birabwa, Daniel Ayen Okello, Pedro Guillermo Suarez, Samuel Kasozi, Raymond Byaruhanga, Deus Lukoye

**Affiliations:** 1 TRACK TB Project, Management Sciences for Health, Bugolobi, Kampala, Uganda; 2 United States Agency for International Development, Kampala, Uganda; 3 Kampala Capital City Authority, Directorate of Public Health and Environment, Kampala, Uganda; 4 Management Sciences for Health, Arlington, Virginia, United states of America; The Ohio State University, UNITED STATES

## Abstract

While old age is a known risk factor for developing active tuberculosis (TB), studies on TB in the population aged 60 years and older (considered elderly in this study) are few, especially in the developing world. Results of the TB prevalence survey in Uganda found high TB prevalence (570/100,000) in people over 65. We focused on treatment outcomes in the elderly to understand this epidemic better. We conducted a retrospective analysis of data from TB facility registers in Kampala City for the period 2014–2015. We analyzed the 2014–15 cohort with respect to age, sex, disease class, patients’ human immunodeficiency virus (HIV) and directly observed therapy (DOT) status, type of facility, and treatment outcomes and compared findings in the elderly (≥60) and younger (<60) age groups. Of 15,429 records, 3.3% (514/15,429) were for elderly patients. The treatment success rate (TSR) among elderly TB patients (68.3%) was lower than that of the non-elderly (80.9%) and the overall TSR 80.5%, (12,417/15,429) in Kampala. Although the elderly were less likely to test positive for HIV than the young (AOR 0.39; 95% CI 0.33–0.48, p<0.001), they had a two-fold higher risk of unfavorable treatment outcomes (AOR 2.14; CI 1.84–2.72, p<0.001) and were more likely to die while on treatment (AOR 1.86; CI 1.27–2.73; p = 0.001). However, there was no statistically significantly difference between treatment outcomes among HIV-positive and HIV-negative elderly TB patients. Compared to the younger TB patients, elderly TB patients have markedly poorer treatment outcomes, although TB/HIV co-infection rates in this age group are lower.

## Introduction

The global burden of the tuberculosis (TB) epidemic in 2016 was estimated at 10.4 million people, much higher than the previous year. Despite the availability of effective TB treatment, TB remains one of the 10 top causes of death globally [1]. Moreover, although TB is known as a disease of younger age groups in countries with high prevalence of the disease [2], its incidence among the elderly has increased over the past three decades [3–5] and its diagnosis in this age group remains a challenge. Unlike in the young, TB in the elderly frequently presents atypically, making its diagnosis difficult; it is often unrecognized until diagnosis is made at autopsy [6], [7]. Currently, global efforts to control TB are focused mainly on key vulnerable populations, such as people living with HIV (PLHIV), people living with diabetes, women, and children, excluding the elderly [8–11]. Targets such as “zero TB deaths among children” [12], [13]are set accordingly. Globally, the elderly population is increasing [11], and old age presents health challenges due to immunosenescence [2], [14] The need to prioritize TB care in the elderly is, therefore, critical for reducing the TB burden and TB transmission to the general population, and addressing TB in the elderly will ultimately contribute to the End TB strategy [15].

Data collection tools for national programs collect TB data on age categories to meet WHO reporting requirements regarding new and relapse patients [16], but cohort data analyses on treatment outcomes involving age disaggregation are not a common practice. We aimed to establish, therefore, the contribution of the elderly (defined as those aged 60 years or more) to the TB burden [17] in Kampala and their treatment outcomes as compared to those of the younger population.

## Study population and methods

### Study design

We studied retrospective data collected routinely from TB registers in 62 Kampala health units during the period 2014–2015. This is routine data collected monthly by the Divisional TB/Leprosy Supervisors for program monitoring in the city. We hypothesized that there was no difference in treatment outcomes between elderly TB patients and non-elderly TB patients seeking care at health facilities in Kampala district.

### Study area

The study was conducted in 62 Kampala City health facilities that report TB cases to the Kampala Capital City Authority (KCCA). Kampala’s population is estimated to be 1,507,080 people [18] with 28,975 (1.9%) of the total population estimated to be people aged 60 years and above [19]. Data from January 1, 2014, to December 31, 2015, from Kampala health unit TB registers were collected and used for this study. For purposes of this study, patients aged 60 years and above were defined as elderly.

### Data management and analysis

We extracted data from the unit TB electronic register [20] into Microsoft Excel (2013) and analyzed the data using Stata version 12.1 (StataCorp, Stata Statistical Software: Release 12, College Station, TX: StataCorp LP; 2011). The electronic register was a pilot electronic database aimed at keeping track of performance of TB patients within the district. It captured patient variables of interest to the TB program by health facility. The patient variables extracted were: age, sex, disease classification (PBC, PCD, EP), type of patient (New, Relapse, Previously lost to follow up, or Failure), HIV status (Positive or Negative), Co-trimoxazole preventive therapy (CPT) uptake status and Antiretroviral Therapy (ART) status for HIV patients, TB treatment model (Directly observed), TB treatment outcome (cured, completed, failed, died, lost to follow up, not evaluated). Cured and completed treatment outcomes were categorized as favorable while the rest were categorized as unfavorable treatment outcomes. The others were. Continuous data were summarized using medians with interquartile ranges, while categorical data were computed as proportions. We presented the data in text, tables, and graphs. In calculating the incidence rate of TB among elderly people, we assumed that all patients registered in KCCA facilities were residents of Kampala District and were registered during the period considered for analysis. Results were presented at p-value = 0.05 level of significance and 95% confidence interval. All registered patients were included. Patients missing any of the variables used in the analysis were excluded from the analysis.

### Ethical considerations

This study involved a review of existing routine TB program data for analysis hence, no ethics approval was sought. The review was planned and guided by KCCA’s Urban Tuberculosis Control Unit, the Directorate of Public and Environmental Health. KCCA reviewed and approved the manuscript for publication in a peer-reviewed journal.

## Results

Of 15,429 patients records accessed, 3.3% (514/15,429) were for TB patients aged 60 years and above, of whom 60.7% (312/514) were male. While the overall mean age was 67.5 years (± SD 7.5 years), among males and females it was 67.1 years (± SD 7.9 years) and 68.2 years (± SD 7.2 years), respectively. Results showed that 75.1% (386/514) of the patients were on DOT during the time of TB treatment (Table 1). All 514 elderly patients had HIV test results, of which 157 (30.5%) were positive. There were more females (35%) living with HIV than males (27.8%). Among PLHIV, uptake of cotrimoxazole preventive therapy (CPT) was 98.1% (154/157), while that of antiretroviral treatment (ART) was 93.0% (146/157).

**Table 1:** Characteristics of elderly TB patients registered during January 2014 to December 2015.

Category	All TB Patients	Treatment Outcomes for All TB Patients			TB Patients ≥60	Treatment Outcomes for TB Patients ≥60			Proportion of Elderly TB Patients among all TB Patients
		Favorable	Unfavorable	P-value		Favorable	Unfavorable	P-value	
Total, *n*	15,429	12,417 (80.5%)	3,012 (19.5%)		514	351 (68.3%)	163 (31.7%)		3.3%
**Sex**									
Male	9,541 (61.8%)	7,669 (80.4%)	1,872 (19.6%)	0.708	312 (60.7%)	220 (70.5%)	92 (29.5%)	0.211	3.3%
Female	5,888 (38.2%)	4,748 (80.6%)	1,140 (19.4%)		202 (39.3%)	131 (64.9%)	71 (35.1%)		3.4%
**Disease Category**									
Extra-pulmonary	2,543 (16.5%)	1,863 (73.3%)	680 (26.7%)	<0.05	95 (18.5%)	52 (54.7%)	43 (45.3%)	0.004	3.7%
PBC	9,311 (60.3%)	7,802 (83.8%)	1,509 (16.2%)		267 (52.0%)	195 (73.0%)	72 (27.0)		2.8%
PCD	3,575 (23.2%)	2,752 (77.0%)	823 (23.0%)		152 (29.5%)	104 (68.4%)	48 (31.6%)		4.2%
**DOT Status**									
On DOT	11,120 (72.1%)	9,143 (82.2%)	1,977 (17.8%)	<0.001	386 (75.1%)	274 (71.0%)	112 (29.0%)	0.03	3.5%
Not on DOT	4,309 (27.9%)	3,274 (76.0%)	1,035 (24.0%)		128 (24.9%)	77 (60.2%)	51 (39.8%)		3.0%
**HIV Results**									
Positive	7,696 (49.9%)	5,806 (75.4%)	1,890 (24.6%)	<0.001	157 (30.5%)	103 (65.6%)	54 (34.4%)	0.445	2.0%
Negative	7,733 (50.1%)	6,611 (85.5%)	1,122 (14.5%)		357 (69.5%)	248 (69.5%)	109 (30.5%)		4.6%

PBC, pulmonary bacteriologically confirmed; PCD, pulmonary clinically diagnosed. 95% Confidence interval, Pearson’s X^2^.

The risk of HIV infection among the elderly was much lower than among their non-elderly counterparts (AOR 0.39; 95% CI 0.33–0.48), and they were less likely to be classified as pulmonary bacteriologically confirmed (PBC) TB patients (AOR 0.65; 95% CI 0.51–0.83). However, being a retreatment, pulmonary clinically diagnosed (PCD), or extra-pulmonary TB patient was not associated with elderly age. See Table 2.

**Table 2:** Comparison of elderly and non-elderly TB patients.

Variable	<60 (%)	≥60 (%)	Crude OR (CI)	P-Value	AOR (CI)	P-Value
HIV positive	7,539 (50.5%)	157 (30.5%)	0.43 (0.36–0.52)	<0.001	0.39 (0.33–0.48)	<0.001
Retreatment	1,202 (8.1%)	38 (7.4%)	0.91 (0.65–1.27)	0.585	0.90 (0.71–1.39)	0.992
PBC	9,044 (60.6%)	267 (52.0%)	0.70 (0.59–0.84)	<0.001	0.65 (0.51–0.83)	0.001
PCD	3,423 (22.9%)	152 (29.6%)	1.41 (1.16–1.71)	<0.001	1.13 (0.87–1.47)	0.352
Extra-pulmonary	2,448 (16.4%)	95 (18.5%)	1.15 (0.92–1.45)	0.214	0.88 (0.67–1.15)	0.352
DOT	10,734(71.97%)	386 (75.1%)	1.17 (0.96–1.44)	0.120	1.19 (0.97–1.47)	0.09
TB unfavorable outcome^a^	2,849 (19.1%)	163 (31.7%)	1.96 (1.62–2.37)	<0.001	2.24 (1.84–2.72)	<0.001

^a^Unfavorable outcome = died, failed treatment, lost to follow-up, or transferred out.

95% Confidence interval. Adjusted for Sex, type of patient, CPT uptake, ART uptake.

The overall treatment success rate among the elderly was 68.3% (351/514), of whom only 36.8% (189/514) completed treatment and 31.5% (162/514) were cured. About 78.1% (274/351) of the elderly whose TB treatment was successful were documented to be on DOT and 29.3% (103/351) of them were HIV positive. Among the cured patients, 81.5% (132/162) had been on DOT and 28.4% (46/162) were HIV positive. Among those who completed treatment, 75.1% (142/189) had been on DOT and 30.2% (55/189) were HIV positive. Of the 163 (31.7%) patients with unfavorable outcomes, 19.8% (102/514) died, 1.6% (8/514) failed on treatment, 6.4% (33/514) were lost to follow-up, and 3.9% (20/514) were not evaluated (had been transferred out and hence no final treatment outcomes were assigned). In this category, 68.7% (112/163) were on DOT and 33.1% (54/163) were HIV positive. Compared to the non-elderly patients, unfavorable treatment outcomes were higher among the elderly (OR 1.96; CI 1.63–2.37; p<0.001). Of the deaths, 53.0% (35/66) occurred within the first two months of treatment, while a cumulative percentage of 75.7% (50/66) occurred within the first four months of treatment. See Table 3.

**Table 3:** Time of death during treatment.

			Month of Death during Treatment								
Age	Total Died	Documented Date of Death	1	2	3	4	5	6	7	8	9
**<60**	1,539	969	366	196	146	93	69	45	34	14	6
**≥60**	102	66	25	10	7	8	7	6	3	0	0
**Total**	**1,641**	**1,035**	**391**	**206**	**153**	**101**	**76**	**51**	**37**	**14**	**6**

606 patient records did not have a documented date of death.

The elderly had a two-fold higher risk of death (AOR 1.86; CI 1.27–2.73; p = 0.001) than the non-elderly. Generally, the elderly were less likely to register a treatment success (OR 0.51; CI 0.42–0.62; p<0.001) and were less likely to be cured (OR 0.57; CI 0.48–0.69; p<0.001). See Table 4.

**Table 4:** Comparison of treatment outcomes between elderly and non-elderly TB patients.

Variable	<60 n (%)	≥60 n (%)	Crude OR (CI)	P-Value	AOR (CI)	P-Value
Treatment success	12,066 (80.9%)	351 (68.3%)	0.51 (0.42–0.62)	<0.001	0.66 (0.48–0.92)	0.015
Cure (PBC only)	6,606 (44.3%)	162 (31.5%)	0.57 (0.48–0.69)	<0.001	0.74 (0.50–1.08)	0.114
Died	1,539 (10.3%)	102 (19.8%)	2.15 (1.72–2.69)	<0.001	1.86 (1.27–2.73)	0.001
Lost to follow-up	818 (5.5%)	33 (6.4%)	1.18 (0.83–1.69)	0.361	0.74 (0.33–1.64)	0.458
Not evaluated	328 (2.2%)	20 (3.9%)	1.80 (1.14–2.85)	0.012	1.36 (0.78–2.36)	0.282
Treatment failure	164 (1.1%)	8 (1.6%)	1.42 (0.69–2.91)	0.335	1.29 (0.56–2.59)	0.640

96% Confidence interval. Adjusted for Sex, disease classification, type of patient, HIV status, CPT uptake, ART status, and treatment model.

Results showed no statistically significantly difference between treatment outcomes among HIV-positive and HIV-negative elderly TB patients. See Table 5.

**Table 5:** Treatment outcomes among elderly Hiv-positive TB patients.

Variable	HIV Positive	HIV Negative	Crude OR (CI)	P-Value	AOR (CI)	P-Value
Treatment success	103 (65.6%)	248 (69.5%)	0.84 (0.56–1.25)	0.386	0.72 (0.17–3.11)	0.660
Cure (PBC only)	46 (29.3&)	116 (32.5%)	0.86 (0.57–1.29)	0.473	0.92 (0.58–1.46)	0.718
Died	40 (25.5%)	62 (17.4%)	1.63 (1.03–2.55)	0.03	1.93 (0.65–5.74)	0.234
Lost to follow-up	6 (3.8%)	27 (7.6%)	0.48 (0.19–1.20)	0.12	0.37 (0.06–1.99)	0.247
Not evaluated	5 (3.2%)	15 (4.2%)	0.75 (0.27–2.10	0.58	0.55 (0.09–3.21)	0.511
Treatment failure	3 (1.91%)	5 (1.4%)	1.37 (0.32–5.81)	0.67	1.8 (0.31–10.3)	0.511

96% Confidence interval. Adjusted for Sex, disease classification, type of patient, HIV status, CPT uptake, ART status, and treatment model.

Extra-pulmonary TB patients had a higher risk of unfavorable treatment outcomes (AOR 1.78; CI 1.04–3.04; p = 0.03), compared to other patient categories such as PCD and PBC. However, the risk of unfavorable treatment outcomes was lower among elderly TB patients who were on DOT (AOR 0.61; CI 0.4–9.33; p = 0.02). See Table 6.

**Table 6:** Risk of unfavorable outcomes of TB Treatment among the elderly by patient category.

Variable	Unfavorable Outcome	Favorable Outcome	OR (95% CI)	P-Value	AOR (95% CI)	P-Value
EP	43 (45.3%)	52 (54.7%)	2.06 (1.30–3.27)	0.002	1.78 (1.04–3.04)	0.034
PBC	72 (27.0%)	195 (73.0%)	0.63 (0.43–0.92)	0.016	0.79 (0.51–1.23)	0.300
PCD	48 (31.6%)	104 (68.4%)	0.99 (0.66–1.49)	0.967	1.26 (0.81–1.95)	0.300
On DOT	112 (29.0%)	274 (71%)	0.62 (0.41–0.94)	0.023	0.61 (0.4–9.33)	0.023
HIV Positive	54 (34.4%)	103 (65.6%)	1.19 (0.80–1.78)	0.387	1.11 (0.74–1.67)	0.616

95% Confidence interval. Adjusted for Sex, type of patient, CPT Uptake and ART status.

Among the elderly, those who were HIV positive had a higher risk of death (AOR 1.62; 95% CI 1.01–2.56), however, risk of death was lower among pulmonary bacteriologically confirmed TB patients (AOR 0.42; 95% CI 0.26–0.66). See Table 7.

**Table 7:** Risk factors associated with death among the elderly TB population.

Variable	Died	OR (95% CI)	P-Value	AOR (95% CI)	P-Value
On DOT	72 (18.7%)	0.75 (0.46–1.21)	0.241	0.76 (0.46–1.25)	0.289
PBC	36 (13.5%)	0.42 (0.27–0.67)	<0.001	0.42 (0.26–0.66)	<0.001
HIV positive	40 (25.5%)	1.62 (1.03–2.55)	0.035	1.61 (1.01–2.56)	0.046
Male	60 (19.2%)	0.91 (0.58–1.41)	0.665	0.90 (0.57–1.41)	0.659

95% Confidence interval. Adjusted for Disease classification, Type of patient, CPT Uptake and ART status.

## Discussion

Our study explored characteristics and treatment outcomes among elderly TB patients in Kampala, Uganda. TB patients aged 60 years and above accounted for nearly 3.3% of the total TB patients notified, which is comparable to the 3.7% contribution of this age group to the total population [18]. We show that the elderly had a two-fold higher risk of unfavorable treatment outcomes than younger TB patients, a finding similar to what was reported in Tamilnadu, South India [21].

Although TB is documented as a significant problem among the elderly [2], information about TB among the elderly in sub-Saharan Africa remains scanty. The data we collected for this study covered all KCCA health facilities reporting to the National TB/Leprosy Program, making our results generalizable to similar urban settings in Uganda and beyond [18]. Our study covered two years, thereby controlling for potential seasonal variations in health care-seeking patterns that might bias the findings. This study is among the few that attempt to explore TB indicators in the elderly population in a resource limited setting. It is interesting to note that, despite the shorter life expectancy of males compared to females [18], the male-to-female ratio among elderly TB patients remains higher, at 3:2, possibly indicating that more men are affected with TB than females. This finding is further confirmed by the Uganda population-based TB prevalence survey, in which the ratio of TB among males compared to females was 4:1 [5], also similar to what is observed elsewhere [21], with elderly patients showing a higher proportion of men compared to non-elderly patients[22].

Other important findings are that the treatment success rate (TSR) among elderly TB patients (68.3%) was lower than that of the non-elderly (80.9%) and significantly lower than the overall TSR (80.5%) in Kampala. In this case, TB in the elderly represents 31.7% of unfavorable treatment outcomes. This disparity poses a special challenge to the National TB/Leprosy Program as it strives to achieve the End TB Strategy target of 75% reduction in TB deaths by 2025 [11] compared to the 2015 baseline.

Although the proportion of the population of the elderly compared with the general population in Uganda is on the decline [18], worldwide the population of the elderly is on the increase [23]. Given age-associated low immunity, the increasing elderly population, especially in high-TB-burden settings, is likely to increase the incidence and prevalence of TB [24], [25]. The persistently small population of the elderly might mean that any changes in the TB epidemic in Uganda will not be attributed to this population. Nevertheless, treatment failures and loss to follow-up among these patients have the potential to propagate the epidemic—just as in younger patients—and should be closely monitored [24].

The high death rate of 19.4% among the elderly as compared to 10.3% among non-elderly populations of TB patients is also a concern and may make the achievement of the WHO End TB Strategy aims and targets difficult in Kampala. That death occurred mainly in the first two months of initiating treatment may reflect delays in seeking health care and late diagnosis. At the moment, mortality audit data to explain this observed high mortality among the elderly are lacking. This observed high mortality might imply that those with TB stayed untreated in the community for a long time [26], thereby promoting transmission of TB to their close contacts, who may develop active TB in the future. Although death will interrupt the transmission chain, the high basic reproductive ratio of *Mycobacterium tuberculosis* will produce many latent infections, which might progress into active TB disease [27]. The high death rate can also be explained by other studies that have reported advanced age as the leading determinant of death among elderly TB patients while on treatment [28]. These deaths can be averted through active case-finding strategies, as demonstrated by recent studies in Kampala [29].

Since most of the deaths (and lower odds of having a favorable outcome) occurred among those not on DOT, this points to challenges in the quality of care provided to the diagnosed TB patients in this age group. Better approaches to diagnosis and treatment, including more emphasis on DOT among the elderly, are needed. If treatment is interrupted due to self-administered treatment, the likelihood of developing multidrug resistance remains high for both patients and their regular contacts [5]. Our findings, however, show a lower loss- to-follow-up rate (6.4%) than findings from other studies (e.g., 18.9% in India) [30], [31].

Our study also showed that elderly pulmonary TB patients are less likely to be bacteriologically confirmed. Failure and delay to confirm TB in such patients operationally contributes to continued TB transmission as well as increased morbidity and mortality. The TB/HIV co-infection rate in the elderly was considerably lower (30.5%) than in the younger population (50.5%). Consequently, we can speculate that the HIV prevalence is higher among the economically productive age group, a position that is confirmed by findings of the Uganda AIDS Indicator Survey [32]. Our findings showed no statistically significantly difference in treatment outcomes between HIV-positive and HIV-negative elderly TB patients. However, other studies have attributed high mortality among HIV-co-infected TB patients to late health care seeking and some life years lost due to HIV, especially for people who do not have access to ART [33], [34].

The study had some limitations. Since we used a record review of TB registers, it was not possible to validate data about any co-morbidities other than HIV, such as diabetes, which may be a risk factor for TB in lower-income countries [35]. Hence we could not assess the extent of the problem of co-morbidities as it relates to mortality. Furthermore, the population of the elderly was only 3.3% compared to 96.7% of the non-elderly population, which might have skewed our findings. We assumed that all the TB patients were from Kampala District, which might not be the case. Our study considered the United Nations working definition of the elderly as those aged 60 years and above[36]. This guided our age categorization for elderly versus non-elderly comparison and may have resulted into a study bias because further categorizations can be made within each of the groups. Finally, this study did not consider TB in the elderly in a rural setting, which might affect generalizability to such populations.

### Implications of findings

Poor TB treatment outcomes in older adults have often been attributed to delayed diagnosis, increased rates of drug-related adverse events, co-morbidities, and overarching poverty[13], [37]. A high death rate was evidenced among the elderly in our study; however, we did not establish the causes of this high death rate from the available data. The health systems needs to confront the challenges of an ageing population and the integrated services required to address their health needs. Our study also makes a case for the need to implement a systematic regular mortality audit among the elderly and all other TB patients in Uganda using recommended TB mortality audit tools [38], the findings of which may help to increase TB case detection and case holding through early diagnosis and improved TB case management. Older adults often act as society’s caregivers, community leaders, and mentors, and play an important role in educating the younger generation [13]. Increasing focus on them as a key population in TB prevention, active case finding, and treatment interventions will not only have positive cascading effects through families, communities, and societies but also demonstrate inclusiveness in health service provision.

## Conclusions

Elderly TB patients have poorer treatment outcomes than the non-elderly despite having lower TB/HIV co-infection rates and high DOT coverage. They are more likely to present with extra-pulmonary TB than the non-elderly populations. Since there was a high death rate among the elderly TB patient, there is need to understand the major causes contributing to this high rate of death, hence the need for further studies to explore the causes of mortality among this group. Such further studies should comprehensively explore the need for existing TB programs to capture additional heath information on elderly TB patients to guide TB programming; specifically, early case finding and case holding to achieve better treatment outcomes. [39]

## Supporting information

S1 File Anonymized data set. (XLSX)
