# Correction: Response to anti-tuberculosis treatment by people over age 60 in Kampala, Uganda

**DOI:** 10.1371/journal.pone.0210769

**Published:** 2019-01-14

**Authors:** The PLOS ONE Staff

## Notice of republication

An incorrect version of S1 File was published in error. The publisher apologizes for this error. This article was republished on December 21, 2018 to correct for this error. Please download this article again to view the correct version.
